# Designer cell therapy for tissue regeneration

**DOI:** 10.1186/s41232-024-00327-4

**Published:** 2024-03-15

**Authors:** Noyuri Zama, Satoshi Toda

**Affiliations:** 1 WPI Nano Life Science Institute (WPI-NanoLSI), Kanazawa University, Kakuma-machi, Kanazawa , 920-1192 Japan; 2 Graduate School of Frontier Science Initiative, Kanazawa University, Kakuma-machi, Kanazawa , 920-1192 Japan

**Keywords:** Cell engineering, Cell therapy, Synthetic receptor, Tissue regeneration, Tissue microenvironment

## Abstract

Cancer cell therapy, particularly chimeric antigen receptor (CAR) T-cell therapy for blood cancers, has emerged as a powerful new modality for cancer treatment. Therapeutic cells differ significantly from conventional drugs, such as small molecules and biologics, as they possess cellular information processing abilities to recognize and respond to abnormalities in the body. This capability enables the targeted delivery of therapeutic factors to specific locations and times. Various types of designer cells have been developed and tested to overcome the shortcomings of CAR T cells and expand their functions in the treatment of solid tumors. In particular, synthetic receptor technologies are a key to designing therapeutic cells that specifically improve tumor microenvironment. Such technologies demonstrate great potential for medical applications to regenerate damaged tissues as well that are difficult to cure with conventional drugs. In this review, we introduce recent developments in next-generation therapeutic cells for cancer treatment and discuss the application of designer therapeutic cells for tissue regeneration.

## Introduction

Cell-based therapeutics, the utilization of cells as a drug, is becoming a reality [1]. In particular, T cells engineered with chimeric antigen receptor (CAR) T cells are used clinically to recognize and eliminate blood cancer cells [2]. While CAR T cells have shown significant success in treating specific acute and chronic leukemia, challenges persist, including on-target/off-tumor side effects, tumor escape due to the loss of target antigen, CAR T-cell exhaustion, and the immunosuppressive cancer microenvironment [3]. To address these issues and expand engineered T-cell therapy to various cancer types including solid tumors, significant efforts have been devoted to designing next-generation engineered T cells. For example, logic gating for CAR activation improves the specificity or broadness of CAR T-cell recognition [4]. Moreover, genetic engineering of CAR T cells to express interleukine-17 (IL-17) and CCL19, which facilitate T-cell proliferation and stimulate immune cell chemotaxis, respectively, resulted in improved survival in a mouse tumor model [5]. To avoid the rapid exhaustion of patient-derived CAR T cells exposed continuously to antigens, induced pluripotent stem cells (iPSCs) have been used to generate CAR T cells with extended lifespans [6, 7]. These are some examples of next-generation CAR T cells; additional examples are described in detail in other review articles [8, 9].

To engineer therapeutic cells, one of the key technologies for maximizing the strength of cell-based therapeutics is the use of synthetic receptors to define the sensing and response of therapeutic cells [10, 11]. For example, in addition to CAR, synthetic cytokine receptors can convert non-physiological ligands into effective cytokines to control immune cell activity [12, 13]. Synthetic adhesion receptors define the orthogonal cell–cell interactions to spatially organize cells into multicellular tissues [14, 15]. Synthetic Notch (synNotch) receptors and synthetic intramembrane proteolysis receptors (SNIPRs) provide a flexible platform for creating novel cell–cell signaling with gene expression control, as described in the following sections [16, 17]. These synthetic receptor technologies allow us to define what factors related to diseases the therapeutic cells should sense and what therapeutic activities and behaviors they should output in response.

## Designer cell therapy

**Fig. 1:**
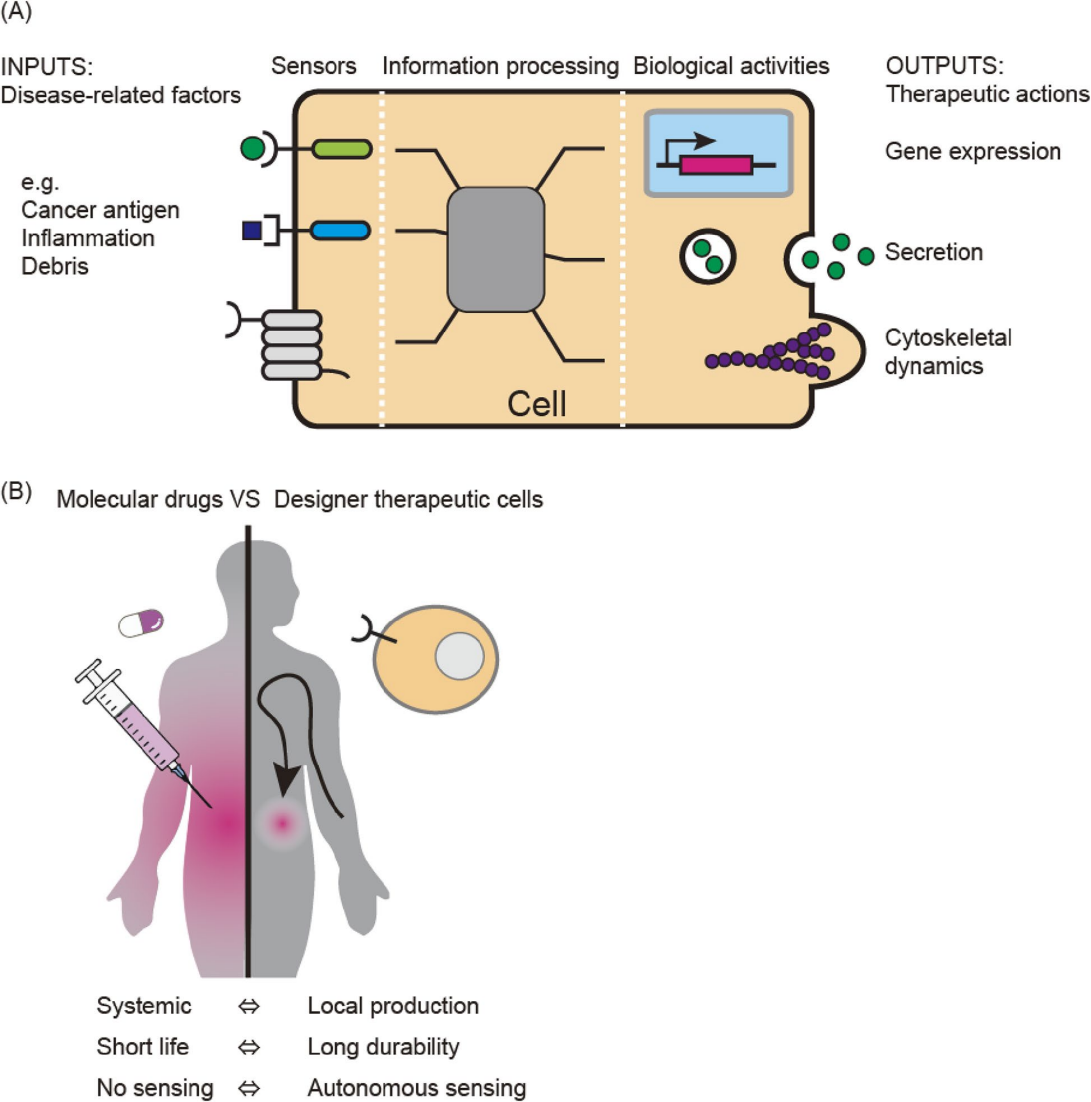
Concept of designer therapeutic cells. **A** Designer therapeutic cells. Cells express receptors that sense environmental molecules. These receptors activate intracellular signaling networks to produce various cellular responses. Such cell systems can be engineered to develop designer therapeutic cells that are programmed to sense disease-related factors and process intracellular signals to output specific therapeutic actions. **B** Advantages of designer therapeutic cells over molecular drugs. While molecular drugs passively diffuse into the body, designer therapeutic cells can sense and reach the disease site, control behaviors such as proliferation and differentiation, and produce user-defined therapeutic factors. These properties enable the specificity and autonomy of therapeutic effects by designer cells (adopted from Lim [1], with modifications)

One of the major differences between therapeutic cells and conventional drugs, such as small compounds and antibodies, lies in the “information-processing ability” of cells [18]. Cells can sense environmental factors using receptors, leading to the activation of intracellular signaling networks, which trigger various output behaviors such as gene expression, secretion, and cytoskeletal rearrangement. Therefore, by programming the environmental factors that cells recognize and the responsive behaviors that cells induce, we can create “designer cells” that can recognize disease sites in the body and perform therapeutic cellular actions against these diseases (Fig. 1A).

In general, when molecular drugs are systemically administered, they spread to a broad area of the body and can cause adverse effects in healthy tissues (Fig. 1B, left). In addition, molecular drugs decay over time after administration; therefore, repeated administration is necessary to maintain drug activity. In contrast, designer cells sense disease-related factors to produce therapeutic activities, leading to disease site-specific effects (Fig. 1B, right). Furthermore, designer cells are activated when symptoms occur and lose their activity when the disease is cured. Thus, the designer cells can deliver therapeutic factors in a disease-specific manner, both spatially and temporally. This higher specificity may enable the safe delivery of potent therapeutic factors to the disease site, which cannot be used for systemic administration owing to their toxicity to healthy tissues. In essence, designer cells can address currently challenging diseases by acting as a “little doctor” that recognizes (diagnoses) diseases and produces (prescribes) therapeutic factors within the body [19]. In addition, this little doctor could ideally control the proliferation and persistence of itself to sustain its therapeutic effects. In the following sections, we use synNotch receptors as an example to discuss how synthetic receptor technologies will transform cell-based therapeutics, and how such technologies can be applied to develop designer therapeutic cells for regenerative medicine.

## Leveraging synNotch receptors for precision cellular sensing and responsiveness

**Fig. 2:**
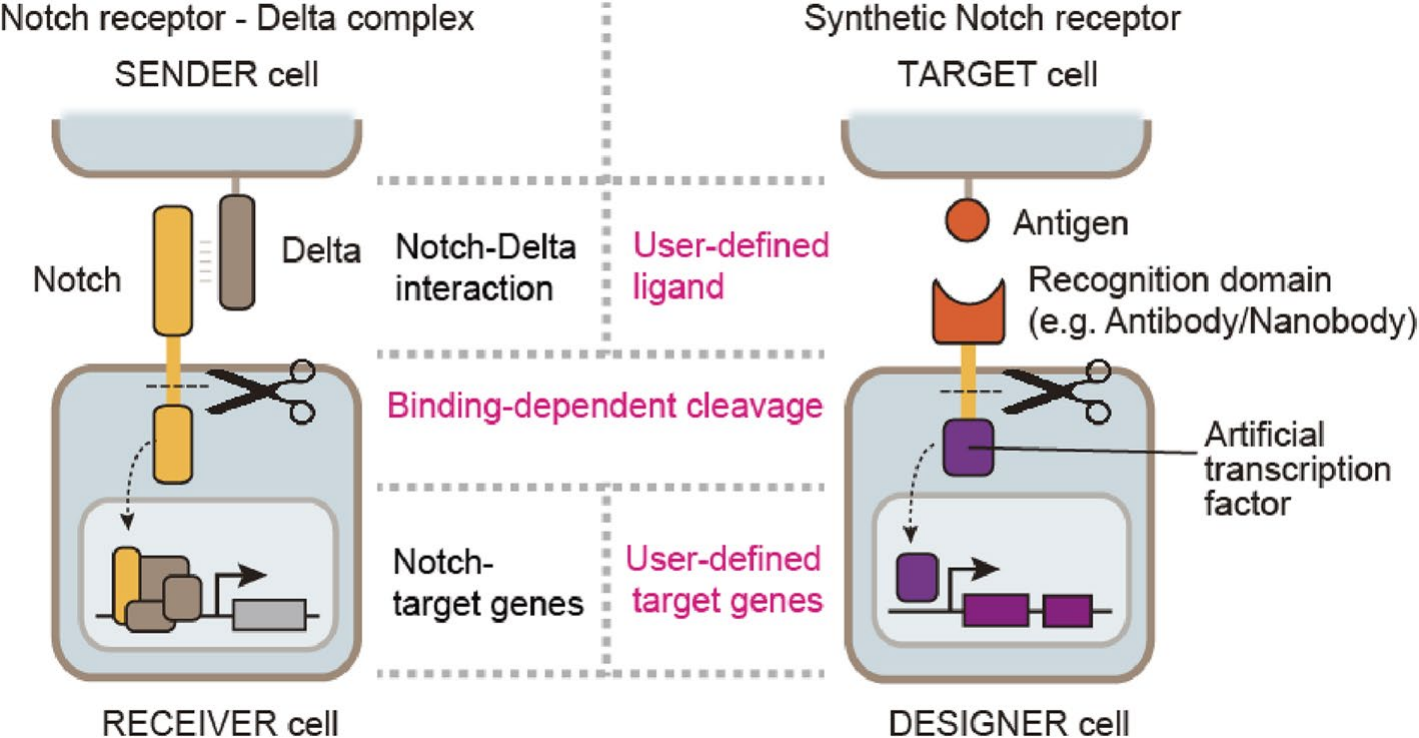
Programming sensing and responsiveness in designer therapeutic cells through synthetic Notch receptors. Notch is a natural signal transduction receptor that recognizes the Delta ligand. The Notch–Delta interaction induces the cleavage of Notch, leading to the release of the Notch intracellular domain. Subsequently, this intracellular domain translocates into the nucleus, where it regulates the expression of Notch target genes. In contrast, the synNotch system contains an extracellular antibody/nanobody domain that recognizes user-defined antigens. SynNotch is also cleaved by antigen recognition; however, it can induce the expression of user-defined target genes by releasing artificial transcription factors

Synthetic receptor technologies have been intensely developed to control diverse cell behaviors with user-defined inputs for both basic science and medical applications. Among them, synNotch is a powerful tool for engineering designer therapeutic cells and has had a huge impact on the field of cell engineering [16]. Notch is a widely used endogenous receptor for cell–cell signaling, especially during development, and is composed of extracellular and intracellular domains that sense and transduce signals, respectively. When Notch binds to its ligand Delta, a mechanical pulling force is exerted on Notch, leading to the exposure of a concealed cleavage site near the cell membrane. This site is subsequently cleaved by an endogenous protease, resulting in the release of the intracellular domain. This domain then translocates to the nucleus and functions as a transcription factor to express Notch-target gene transcription (Fig. 2, left). In contrast, synNotch is a synthetic form of Notch, which is equipped with the desired antibody/nanobody for the extracellular domain and an artificial transcription factor for the intracellular domain (Fig. 2, right). By transducing the target gene plasmid together with the synNotch-expressing plasmid into cells, we can build a cell that can induce user-defined target genes in response to antigen–antibody interactions on the cell surface.

However, one limitation of the synNotch system is that soluble diffusible antigens cannot activate synNotch. To activate the synNotch system, a mechanical pulling force is required from the antigen tethered to a rigid surface, such as cell membrane and plastic plate. Therefore, the original synNotch system lacks the capability to engineer designer cells that can sense disease-related soluble factors, such as inflammatory cytokines and harmful metabolites. Recently, we developed a diffusible synNotch system that senses soluble antigens by first trapping the soluble antigen on the cell surface and presenting it to synNotch for signal transduction [20]. This system enables designer cells to sense disease-related soluble factors and induce therapeutic responses. Because the modularity of the synNotch system enables flexible design of signal input and output, it is expected that synNotch can be widely used to develop designer therapeutic cells that sense and respond to disease-related cells and molecules.

## Empowering next-generation CAR T cells equipped with the synNotch circuit to overcome the immunosuppressive tumor microenvironment

**Fig. 3:**
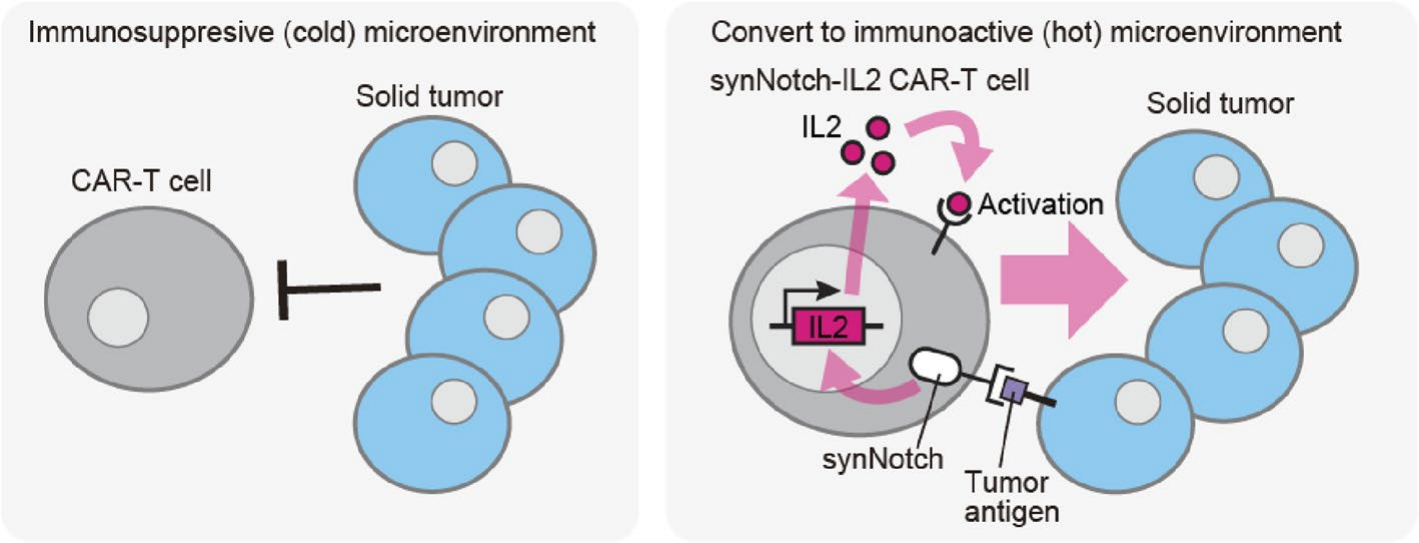
Next-generation CAR T cells with synNotch circuit for targeting immune-suppressive solid tumors. The immunosuppressive (cold) tumor microenvironment around solid tumors blocks CAR T-cell functions. SynNotch circuit–engineered CAR T cells, designed to detect tumor antigens and induce IL-2, can be locally activated with autocrine IL-2 signaling at solid tumor sites. This local activation forms immuno-active (hot) microenvironment to facilitate increased infiltration into solid tumors, which allows successful elimination of solid tumors in a mouse model

Various attempts have been made to develop next-generation CAR T cells equipped with the synNotch system to overcome the current challenges of conventional CAR T-cell therapy [21–25]. One of the major challenges in the application of CAR T-cell therapy is the treatment of solid tumors in an immunosuppressive tumor microenvironment. To transform the immunosuppressive (cold) environment into an immune-active (hot) environment conducive to robust CAR T-cell activity, a synNotch circuit was incorporated into CAR T cells [26]. This circuit induces interleukin-2 (IL-2) in response to cancer antigens. IL-2, a potent immune-stimulator, significantly influences T-cell activation and proliferation, which is one of the promising factors for overcoming the immunosuppressive microenvironment. However, the systemic administration of pro-inflammatory cytokines affects immune cells throughout the body, posing a risk of side effects on healthy tissues [27, 28]. Also, the IL-2 paracrine signaling around IL-2-secreting cells is sharply reduced in the presence of IL-2 consumer cells [29]. Thus, to deliver IL-2 specifically to tumor sites, the IL-2-inducing synNotch circuit was developed and coupled with CAR T cells (Fig. 3). This synNotch–IL-2 circuit allowed CAR T cells to produce IL-2 in a limited manner at the tumor site and activate themselves in an autocrine manner, which helped to overcome the immunosuppressive environment. As a result, synNotch–IL-2 CAR T cells promoted infiltration into the target tumor and improved the efficiency of cancer cell elimination and survival in mouse tumor models of pancreatic cancer and melanoma. This result highlights the potential of designer cells with a synNotch circuit to safely deliver therapeutic factors to specific tissues.

## Designer cell therapy for tissue regeneration

The synNotch circuit empowers designer therapeutic cells to eliminate tumor cells with high efficiency and safety, as described above. Many studies have focused on cancer treatment so far, but the strength of designer therapeutic cells equipped with synthetic receptors is that they can change their behaviors in response to their environment. Therefore, we envision that designer therapeutic cells can be applied not only to cancer but also to many other intractable diseases [30–32]. While designer therapeutic cells have been engineered to eliminate excess unnecessary cells like tumors, they can also be programmed to proliferate necessary cells which are lacking due to tissue damage and injury. Many intractable diseases cause serious tissue damage, including chronic inflammation and degenerative diseases [33, 34]. In addition to tissue damage, the tissue microenvironment, known as the stem cell niche, is disrupted by such diseases. Therefore, the repairment of the niche would also help to drive stem cell proliferation and tissue regeneration [35]. In such scenarios, designer therapeutic cells that specifically recognize damaged tissues and provide therapeutic factors to remove the cause of tissue damages and recover the stem cell niche could present a novel approach to treating intractable diseases with tissue damage.

**Fig. 4:**
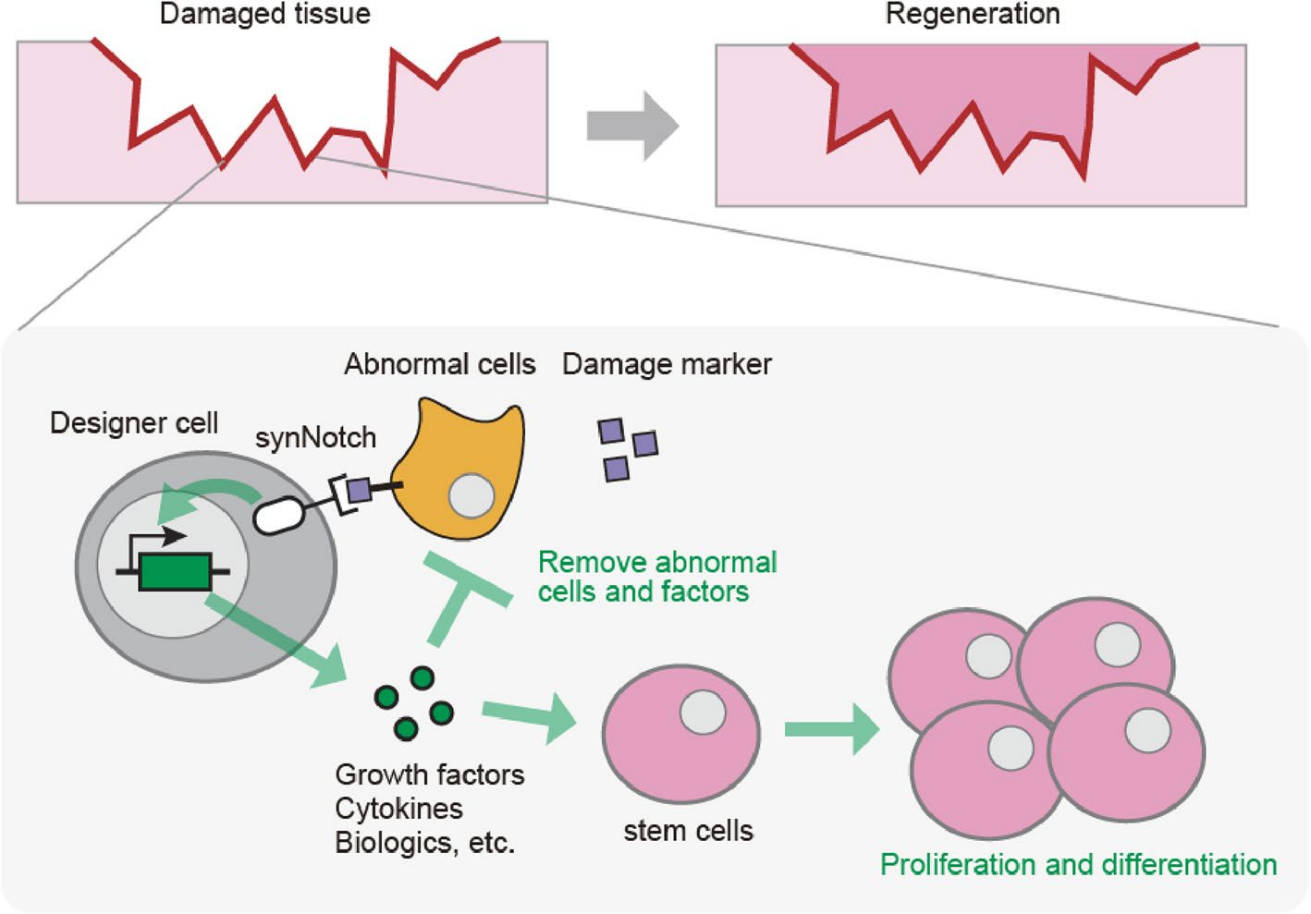
Designer therapeutic cells for tissue regeneration. Designer therapeutic cells are engineered using synNotch to recognize abnormal cells or damage markers specific to damaged tissues. Designer cells then produce a combination of therapeutic factors, including growth factors, cytokines, and biologics, which have different functions to support tissue regeneration by removing damage-related abnormal factors and inducing the proliferation and differentiation of stem cells. Using designer therapeutic cells, potent therapeutic factors can be delivered specifically to damaged tissues with less toxicity to healthy tissues

The synNotch circuit may also aid in the design of therapeutic cells for tissue regeneration (Fig. 4). In many cases, damaged tissues are accompanied by chronic inflammation and cell death, resulting in the recruitment of abnormal cells, such as excessive immune cells and fibroblasts, and the release of cytokines and cell debris. While these factors contribute to tissue damage and disrupt the microenvironment for regeneration, they can function as antigens specific to damaged tissues [36]. By programming cells to recognize damaged tissue-specific antigens and inducing factors to support tissue regeneration using the synNotch system, it will be possible to design therapeutic cells for tissue regeneration. There are many types of candidate target genes that can be induced by synNotch to promote tissue regeneration. For example, growth factors that induce the proliferation of stem cells and anti-inflammatory factors that extinguish inflammation are obviously important [37–39]. In addition to natural signaling molecules, biologics are helpful in specifically blocking signaling molecules that cause tissue damage and chronic inflammation. Thanks to the flexibility of the synNotch system, designer therapeutic cells can be equipped with any genetically encoded therapeutic molecules with tunability on their induction level [40]. They can also induce multiple therapeutic genes by using multiple inducible promoters or polycistronic expression system such as internal ribosome entry site (IRES) or 2A peptides [41, 42]. In addition, the local production of therapeutic factors by designer therapeutic cells would generate their concentration gradient at the damaged tissue and minimize their adverse effects on outside healthy tissues. Engineering designer cells to induce a combination of multiple therapeutic target genes by synNotch will enable designer cells to remove the cause of diseases, repair the tissue microenvironment, and induce the proliferation and differentiation of stem cells at the same time. In instances where endogenous stem cells are lost because of tissue damage, co-transplantation of designer therapeutic cells alongside stem cells holds potential for rebuilding tissues. After the completion of tissue regeneration, the designer cells revert to an inactive state due to the loss of damaged tissue-specific antigens. This transition is crucial for ensuring safety by preventing excessive cell proliferation and the formation of tumors. Such temporal control is also a strong point of designer therapeutic cells with synNotch circuit, which has not been achieved by the therapeutic cells that constitutively express therapeutic factors.

## Future perspective

Despite the potential for designing therapeutic cells in tissue regeneration discussed above, several challenges must be addressed for their practical implementation. First, the origin of these designer therapeutic cells requires exploration. Similar to CAR T cells, T cells emerge as strong candidates because of their ease of injection into the body, ability to circulate throughout the entire body, and capacity to expand in response to specific antigens. Additionally, cell types such as macrophages and mesenchymal cells, capable of residing and moving within target tissues, present as potentially valuable alternatives. Second, as tissue regeneration takes time, designer cells must persist for a long time once transplanted into patients. If designer cells quickly disappear or become exhausted, it becomes necessary to repeatedly administer designer cells to patients, which is unrealistic. Ideally, once designer cells are injected, they maintain a constant population size to support tissue regeneration for several years. For this purpose, designer cells should be equipped with synthetic homeostatic circuits to control their population size and avoid uncontrolled proliferation [43]. Finally, a deeper understanding of endogenous cell–cell communication during tissue development, wound healing, and disease pathology in vivo is necessary for the precise design of therapeutic cell actions to drive efficient regeneration. To overcome these hurdles, the designer therapeutic cells harboring tissue repairing functions will become increasingly useful for treating various types of intractable diseases that are difficult to cure with current medicines.

## Data Availability

Not applicable.
